# In Vivo and In Vitro Comparison of the DPP-IV Inhibitory Potential of Food Proteins from Different Origins after Gastrointestinal Digestion

**DOI:** 10.3390/ijms23158365

**Published:** 2022-07-28

**Authors:** Léa Fleury, Barbara Deracinois, Camille Dugardin, Alice B. Nongonierma, Richard J. FitzGerald, Christophe Flahaut, Benoit Cudennec, Rozenn Ravallec

**Affiliations:** 1 UMR-T 1158, BioEcoAgro, University of Lille, 59650 Lille, France; lea.fleury.etu@univ-lille.fr (L.F.); barbara.deracinois@univ-lille.fr (B.D.); camille.dugardin@univ-lille.fr (C.D.); christophe.flahaut@univ-artois.fr (C.F.); 2 Department of Biological Sciences, University of Limerick, V94 T9PX Limerick, Ireland; alice.nongonierma@kerry.com (A.B.N.); dick.fitzgerald@ul.ie (R.J.F.)

**Keywords:** DPP-IV activity, type 2 diabetes, bioactive peptides, dietary proteins, in vitro gastrointestinal digestion, peptidomics

## Abstract

Dipeptidyl-peptidase IV (DPP-IV) plays an essential role in glucose metabolism by inactivating incretins. In this context, food-protein-derived DPP-IV inhibitors are promising glycemic regulators which may act by preventing the onset of type 2 diabetes in personalized nutrition. In this study, the DPP-IV-inhibitory potential of seven proteins from diverse origins was compared for the first time in vitro and in vivo in rat plasma after the intestinal barrier (IB) passage of the indigested proteins. The DPP-IV-inhibitory potentials of bovine hemoglobin, caseins, chicken ovalbumin, fish gelatin, and pea proteins were determined in rat plasma thirty minutes after oral administration. In parallel, these proteins, together with bovine whey and gluten proteins, were digested using the harmonized INFOGEST protocol adapted for proteins. The DPP-IV half-maximal inhibitory concentration (IC_50_) was determined in situ using Caco-2 cells. The DPP-IV-inhibitory activity was also measured after IB passage using a Caco2/HT29-MTX mixed-cell model. The peptide profiles were analyzed using reversed-phase high-performance liquid chromatography tandem mass spectrometry (RP-HPLC-MS/MS) with MS data bioinformatics management, and the IC_50_ of the identified peptides was predicted in silico. The in vitro and in vivo DPP-IV-inhibitory activity of the proteins differed according to their origin. Vegetable proteins and hemoglobin yielded the highest DPP-IV-inhibitory activity in vivo. However, no correlation was found between the in vivo and in vitro results. This may be partially explained by the differences between the peptidome analysis and the in silico predictions, as well as the study complexity.

## 1. Introduction

Over the past decades, type 2 diabetes (T2D) has become a major public health concern. In 2021, the International Diabetes Federation reported that 537 million people had diabetes worldwide. This figure is predicted to rise to 643 million by 2030, with T2D accounting for 90% of cases. This pathology affects more and more young people [1]. In addition to the social impact, T2D has a significant economic impact on countries and health systems. The annual global health expenditure on diabetes is about €882 billion, representing an increase of 316% over the past 15 years [2]. Medication, the treatment of complications, and the management of disability or premature death all represent a considerable cost to individuals and governments.

T2D is caused mainly by excess weight and a lack of physical activity, leading to a dysregulation of glucose homeostasis. This leads to a prolonged excess of plasma glucose concentration induced by impaired insulin secretion, and/or insulin resistance in peripheral tissues (liver, muscle) [3,4]. In the long term, hyperglycemia causes the onset of severe and even fatal complications, such as cardiovascular diseases (stroke, myocardial infarction, etc.), neurodegeneration, nephropathies, and eye diseases, etc. [5]. T2D can be controlled by lifestyle and dietary measures. However, when it can no longer be managed, a restrictive and sometimes invasive drug treatment (the injection of insulin) may be required, which has undesirable effects [6]. The search for alternative treatments to metformin, the gold standard drug in the management of T2D, focuses on (i) the inhibition of sodium/glucose co-transporter 2 (SGLT2) leading to the reduced renal reabsorption of glucose, and (ii) the inhibition of dipeptidyl-peptidase IV (DPP-IV), which indirectly improves insulin secretion [7]. DPP-IV is an enzyme that cleaves and inactivates incretin hormones such as glucagon-like peptides 1 (GLP-1) and gastric inhibitory polypeptide (GIP), which are responsible for insulin secretion [3]. GLP-1 and GIP are secreted into the bloodstream by intestinal cells after contact with nutrients. The ingestion of monosaccharides, long-chain fatty acids, proteins and certain amino acids stimulates GLP-1 secretion [8]. These two hormones stimulate insulin secretion by pancreatic beta cells [9]. In this context, DPP-IV inhibitors promote insulin secretion in a glucose-dependent manner by extending the half-life of the incretins [10,11]. DPP-IV inhibitors have the advantage of not causing weight gain or hypoglycemia. They also have a long-lasting anti-hyperglycemia effect and a shallow drug interaction risk compared to other T2D medications [4,12]. Therefore, the search for novel DPP-IV-inhibitory molecules is an interesting strategy for the management of T2D.

Several studies in humans and rodents have shown that dietary proteins can regulate glucose homeostasis by lowering plasma glucose levels [13,14] and increasing insulin sensitivity [3]. Furthermore, it has been shown in humans that dietary protein improves glucose homeostasis by stimulating insulin and incretin secretion [15,16]. Dietary proteins can also improve glucose tolerance in short-term conditions in diabetics [17,18]. However, the mechanism(s) of action behind the role of dietary proteins in glucose homeostasis is not yet fully understood. Currently, researchers are focusing on mechanisms involving the direct effect of protein-derived peptides and amino acids on (i) the stimulation of insulin secretion by pancreatic cells [19], (ii) the stimulation of incretin secretion (GIP, GLP-1) and cholecystokinin by enteroendocrine cells [20,21,22,23], (iii) the inhibition of intestinal glucose uptake [24], and (iv) the inhibition of the enzymes involved in the regulation of the glucose level (i.e., DPP-IV and α-glucosidase) [25]. As a consequence, many studies have focused on natural DPP-IV inhibitors originating from the hydrolysis of dietary proteins [26,27,28,29,30,31,32,33,34].

In this context, this study compared seven different dietary protein substrates originating from animal (cow, chicken and fish) and plant (pea and wheat) sources in terms of their ability to inhibit DPP-IV activity. The aim was to determine the potential of peptides derived from these protein substrates to prevent and manage T2D in the framework of personalized nutrition. First, this study compared the potential ability of the different proteins to inhibit plasmatic DPP-IV in rat. In parallel, the ability of the peptides, generated during in vitro digestion of the seven proteins, to cross the intestinal barrier (IB) and to inhibit DPP-IV activity was studied with a cocultured cell-based in vitro IB model. Reversed-phase high performance liquid chromatography tandem mass spectrometry (RP-HPLC-MS/MS) was employed to analyze and identify peptides able to cross the in vitro IB model system. Furthermore, a quantitative structure-activity relationship (QSAR) employed was used to predict their DPP-IV half-maximal inhibitory concentration (IC_50_).

## 2. Results

### 2.1. Characterization of the Protein Content

**Figure 1:**
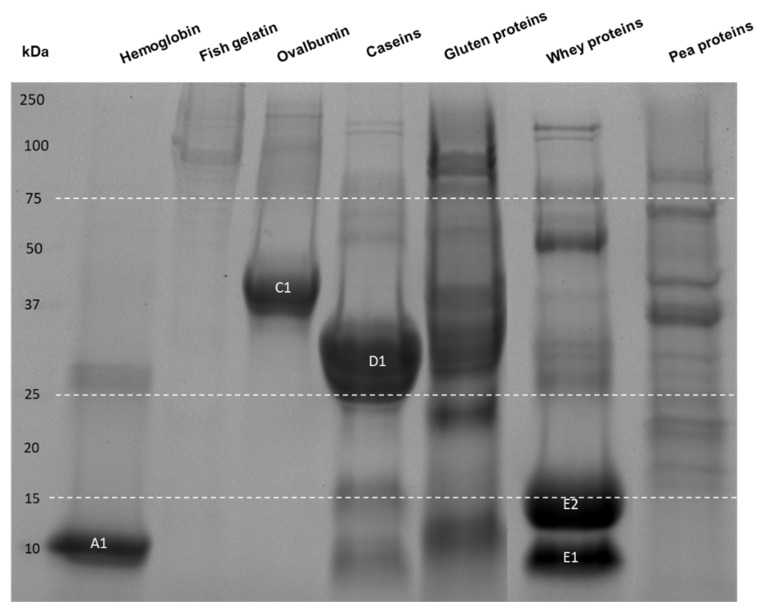
Apparent molecular mass distribution of the seven raw protein samples determined by SDS-PAGE. The amount of solubilized protein powder loaded was 17.4 µg for the hemoglobin and ovalbumin proteins; 69.6 µg for the caseins, whey, fish gelatin and pea proteins; and 522 µg for the gluten proteins. A1: α- and β-subunits of hemoglobin, C1: ovalbumin, D1: caseins, E1/E2: α-lactoglobulin/β-lactoglobulin.

The protein profile of the seven different dietary protein substrates was determined by SDS-PAGE (Figure 1) and a proteomic approach (Table 1).

For the proteomic approach, the proteins were reduced, alkylated, hydrolyzed by trypsin, and subjected to an RP-HPLC-MS/MS analysis; the bioinformatic treatment of the MS-data was used to identify the peptides and deduce the parent proteins (Table 1).

The hemoglobin and ovalbumin protein samples were the purest in terms of protein content. Indeed, very few peptides other than the α and β-subunits of hemoglobin or ovalbumin were identified by MS. The SDS-PAGE confirmed these results, showing two main bands (A1 for the α and β-subunits of hemoglobin and C1 for ovalbumin). The proteomics analysis of the fish gelatin sample allowed the identification of fibrillar and α type 1 collagen with only 16 identified peptides. In addition, the electrophoretic analysis of this sample was made difficult by the poor quality of the migration, probably due to high-molecular-weight gelatin. Regarding the milk protein samples, the caseins and whey proteins each contained the same proteins but in different abundance, either more caseins (D1) or more whey proteins (E1 and E2), which was in line with their process of extraction (see the M&M section). The plant protein samples were a mixture of proteins like glutenin and gliadin for the gluten sample; legumin, vicilin, convicilin, provicilin, lectin, and albumins for the pea protein sample; and other protein constituents such as antinutritional factors or enzymes, as revealed by the proteomic analysis and by the presence of numerous distinct bands at different molecular masses in the gel electrophoretogram.

### 2.2. Protein Ingestion Inhibits Plasma DPP-IV Activity in Rats

**Figure 2:**
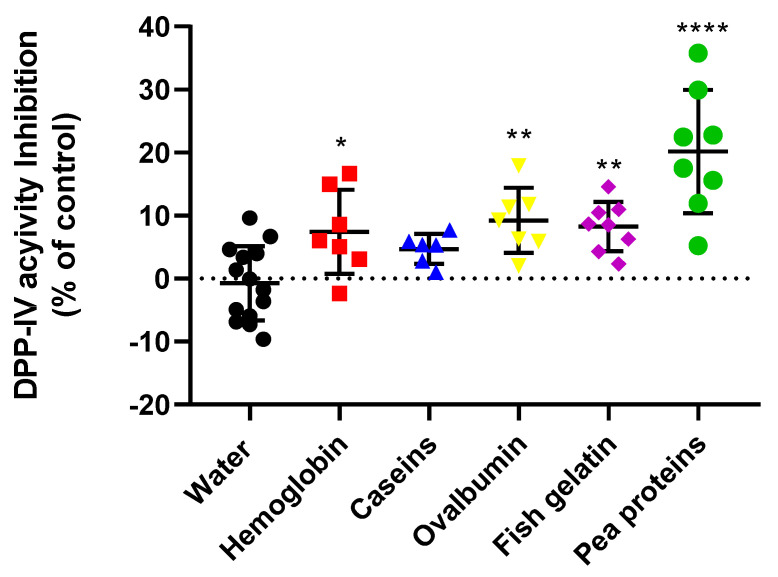
Dietary proteins decrease plasma DPP-IV activity after protein ingestion in rats. Overnight-fasted rats received either water (control) or a protein solution (hemoglobin, ovalbumin, caseins, pea proteins or fish gelatin) at a dose of 1 g·kg^−1^ body weight by oral gavage 30 min before the collection of the blood samples. DPP-IV activity was evaluated by calculating the percentage of the DPP-IV activity inhibition of each protein relative to the control group (water). One–Way ANOVA analysis (Dunnett’s multiple comparison test) was performed to calculate statistical differences (* *p* < 0.05; ** *p* < 0.01; **** *p* < 0.0001) compared to the water control group (n = 8).

The effect of hemoglobin, caseins, ovalbumin, fish gelatin, and pea proteins on DPP-IV activity was first evaluated in vivo in rat plasma. The different protein samples (1 g·kg^−1^) were orally administrated, and blood samples were collected 30 min later in order to measure the plasma DPP-IV activity (Figure 2).

All proteins except caseins significantly decrease plasma DPP-IV activity. Pea proteins were the most efficient, and decreased DPP-IV activity by 20.2% (*p* < 0.0001). Ovalbumin and fish gelatin decreased it by 9.2 and 8.2% (the *p* values were 0.0054 and 0.0094), respectively, and hemoglobin decreased rat plasma DPP-IV activity by 7.4% (*p* = 0.0297).

### 2.3. Digested Proteins Decrease DPP-IV Activity In Vitro at the Enterocyte Level and after IB Passage

**Figure 3:**
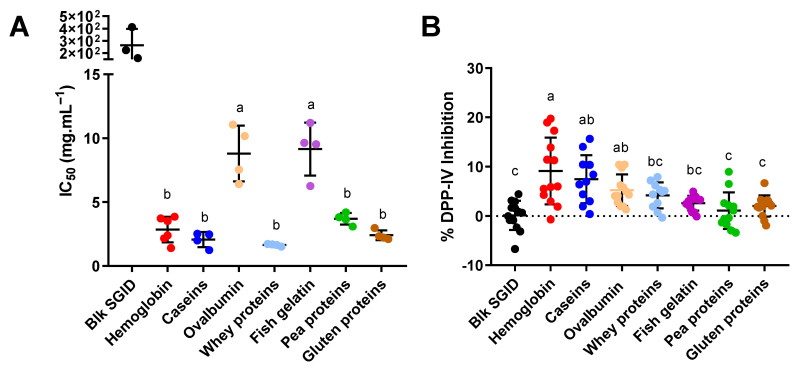
Digested proteins exert different DPP-IV-inhibitory activity in intestinal cells (Caco-2) before (**A**) and after (**B**) simulated IB passage. (**A**) The IC_50_ of digested proteins or blk SGID on DPP-IV activity was determined in live Caco-2 cells. The values are the mean of repeated measurement (N > 3). One–Way ANOVA analysis with Tukey’s post-hoc test (*p* < 0.0001) was performed. (**B**) the DPP-IV-inhibitory activity induced by the basal media after 1.5 h incubation with the digested proteins (31.25 mg·mL^−1^). The values are the means of 12 repeated measurements (N = 4 in triplicate). Statistical analysis was performed using one–Way ANOVA analysis with Tukey’s post-hoc test (*p* < 0.05). The means without a common letter are statistically different.

Dietary proteins were digested following simulated gastrointestinal digestion (SGID) using the INFOGEST protocol [35] adapted to proteins alone [36]. The digested proteins and blank digestion (blk SGID) samples were assayed for their ability to inhibit DPP-IV activity at the enterocyte level using live Caco-2 cells. The results showed that digested hemoglobin, caseins, whey, pea and gluten proteins strongly inhibit intestinal cell DPP-IV activity, with an average IC_50_ value of 2.67 ± 0.7 mg·mL^−1^ (Figure 3A). Digested ovalbumin and gelatin were approximately four times less efficient in the inhibition of DPP-IV activity, with an average IC_50_ value of 9.85 ± 1.25 mg·mL^−1^. Nevertheless, all of the tested digested proteins significantly inhibit DPP-IV activity compared to blk SGID, which was used as control (*p* < 0.0001).

Then, the fraction of the digested proteins collected at the basal side of the IB was assessed for its ability to inhibit DPP-IV in vitro. Caco-2/HT29-MTX were incubated for 1.5 h with digested proteins in the apical compartment. Basal media were recovered at the end of the incubation time in order to test their ability to inhibit DPP-IV activity. The results showed that all of the basal media seem to decrease DPP-IV activity, but only digested hemoglobin, caseins and ovalbumin significantly inhibited DPP-IV activity compared to blk SGID (Figure 3B). The basal media obtained after incubation with digested hemoglobin decrease DPP-IV activity by approximately 9.12% (*p* < 0.001), caseins decrease it by 7.47% (*p* = 0.0003), and ovalbumin decreases it by 5.25% (*p* = 0.020).

### 2.4. Effect of Digested Proteins on Intestinal DPP-IV Gene Expression

**Figure 4:**
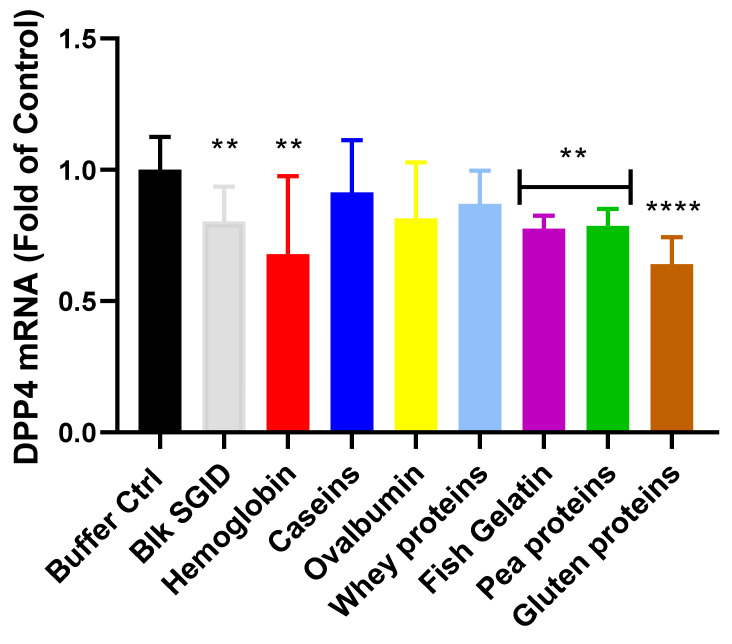
Digested proteins inhibit DPP-IV gene expression after 24 h in a Caco-2/HT29-MTX coculture. The digested protein samples (5 mg·mL^−1^) were incubated with Caco-2/HT29-MTX cocultured cells for 24 h before DPP-IV gene expression measurement by qPCR normalized to HPRT1. The values are the means of nine repeated measurements (N = 3 in triplicate). The statistical analysis was performed using Kruskal–Wallis analysis with Dunn’s post-hoc test (** *p* < 0.01; **** *p* < 0.0001).

In order to investigate the effect of dietary proteins on DPP-IV gene expression at the intestinal level, in vitro digested proteins were incubated for 4, 12 and 24 h with the Caco-2/HT29-MTX cocultured cells. No significant differences were seen between the groups incubated for 4 and 12 h and the control. However, significant differences were observed after 24 h incubation (Figure 4). All of the digested proteins, except the dairy proteins and ovalbumin, inhibited DPP-IV mRNA expression. Digested gluten proteins decreased DPP-IV mRNA expression by 36% (*p* < 0.0001), while hemoglobin, fish gelatin and pea proteins, as well as blk SGID, decreased DPP-IV mRNA expression by about 25% (*p* < 0.01) compared to the buffer Ctrl (Figure 4). However, the digested proteins did not induce any significant mRNA expression changes compared to blk SGID (*p* > 0.05).

### 2.5. Effect of Digested Proteins on IB Integrity and Permeability

During the in vitro IB permeation experiments, the transepithelial electrical resistance (TEER) was measured before and after incubation with the digested proteins. In parallel, the IB permeability was determined at the end of the experiment by measuring the apparent permeability coefficient (P_app_) using lucifer yellow (LY) (Table 2).

The apparent permeability coefficient (P_app_) in cm·s^−1^ was determined with lucifer yellow. The absolute permeability values are the means of repeated measurement (n ≥ 12; n = 3 in quadruplicate). Statistical analysis was performed using Kruskal–Wallis analysis with Dunn’s post-hoc test (** *p* < 0.01; **** *p* < 0.0001) to compare the digested samples to the buffer control. The transepithelial electrical resistance (TEER) of the Caco-2/HT29-MTX coculture for 21 days was measured before and after contact (1.5 h) with the digested proteins (31.25 mg·mL^−1^). The values are the difference between the final and initial TEER from an average of repeated measurements (N = 4). Statistical analysis was performed using two–Way ANOVA analysis with Sidack’s multiple comparison post-hoc test (** *p* < 0.01; **** *p* < 0.0001) to compare the digested samples to the buffer control.

The blk SGID, digested ovalbumin and digested fish gelatin significantly decreased the permeability of the Caco-2/HT29-MTX cell monolayer compared to the buffer control (*p* < 0.0001, *p* = 0.0063, and *p* = 0.0025). On the other hand, the digested hemoglobin, casein, and whey decreased TEER by 34, 26 and 42%. TEER is a marker of membrane integrity. However, the TEER value remains within acceptable limits, as the lowest value obtained was 333 Ω for digested whey proteins after 1.5 h.

### 2.6. Impact of Digested Proteins on Tight Junction (TJ) Protein Gene Expression

**Figure 5:**
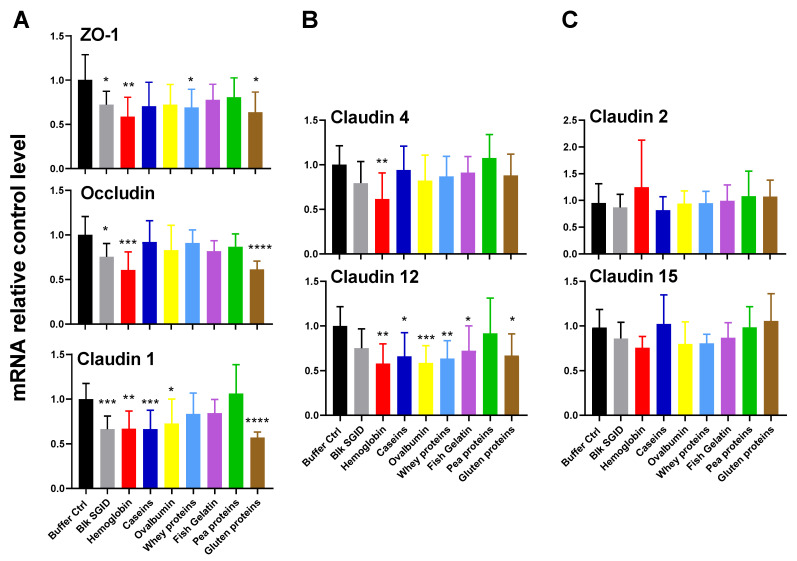
Digested proteins modulate TJ protein gene expression in vitro. (**A**) ZO-1, Occludin, and Claudin 1; (**B**) Claudin 4 and Claudin 12; (**C**) Claudin 2 and Claudin 15 mRNA relative expression levels normalized to HPRT1 in Caco-2/HT29-MTX cocultured cells incubated for 24 h with digested proteins (5 mg·mL^−1^). The relative mRNA levels were expressed in the fold of the buffer control level value. For ZO-1, Claudin 1, Claudin 4, Claudin 2 and Claudin 15, the statistical analysis was performed using two–Way ANOVA analysis with Dunnett’s multiple comparison post-hoc test. For Occludin and Claudin 12, Kruskal–Wallis analysis was performed, followed by Dunn’s multiple comparison post-hoc test to compare digested protein samples to the Buffer Ctrl group (* *p* < 0.05; ** *p* < 0.01; *** *p* < 0.001; **** *p* < 0.0001).

We investigated the effect of the digested proteins on gene expression related to membrane integrity and molecule passage. The studied genes were Zonula Occludens-1 (ZO-1), Occludin, and Claudins 1, 4, and 12 for integrity, and Claudins 2 and 15 for permeability (Figure 5).

The digested samples tended to decrease the gene expression of TJ protein genes, particularly genes encoding ZO-1, Occludin, and Claudin 1 and 12. Digested hemoglobin decreased the gene expression of all of the TJ proteins (*p* < 0.01) except for Claudin 2 and 15. None of the digested proteins influenced the expression of genes encoding Claudins 2 and 15. Digested gluten caused the most significant drop in gene expression of Occludin and Claudin 1, by 39 and 44%, respectively (*p* < 0.0001), and significantly decreased Claudin 12 expression (*p* = 0.03). Digested caseins and ovalbumin significantly reduced ZO-1 and Claudin 1 and 12 gene expression (0.05 < *p* < 0.001), and digested whey protein significantly decreased ZO-1 and Claudin 12 gene expression (*p* = 0.006 and *p* = 0.005, respectively). Blk SGID also significantly reduced the gene expression of ZO-1, Occludin, and Claudin 1 (*p* = 0.011, *p* = 0.028, and *p* = 0.0005, respectively). Finally, contrary to the other digested proteins, pea proteins had no significant effect on TJ protein gene expression.

### 2.7. Peptide Identification after Simulated IB Passage and the In Silico Prediction of Their DPP-IV-Inhibitory Activity

**Figure 6:**
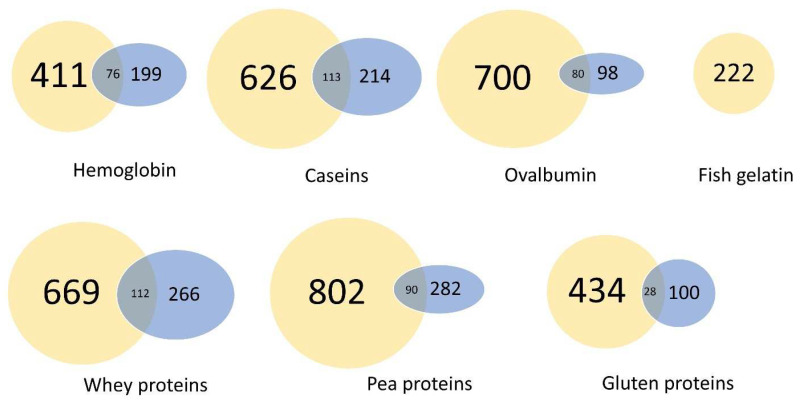
Peptides released during the digestion of dietary proteins can cross the IB in vitro. Peptide distribution in the Caco-2/HT29-MTX cocultured IB model after 1.5 h incubation with digested dietary proteins (31.25 mg·mL^−1^). The number of different peptides identified (de novo included) at the apical (yellow), basolateral (blue), and both sides (grey) are indicated for each digested protein.

Following the in vitro IB passage experiment simulated by the Caco-2/HT29-MTX coculture, the intact peptides which were resistant to digestion and those cleaved by the peptidases from the IB were identified in the apical and basolateral compartments by RP-HPLC-MS/MS and the bioinformatic treatment of the MS-data. Figure 6 represents the number of identified peptides for each digested protein sample in the apical, in the basolateral, and in both compartments. In total, 4624 peptide sequences were identified (by the interrogation of the databases and by the spectral reading of the de novo with an average local confidence (ALC) score > 80%) in all of the sources combined. On average, 3.6 times fewer peptides were identified in the basolateral than in the apical compartments. Around 11% of the identified peptides in the basal compartment were able to cross the IB while remaining intact. At the same time, 14% of the new peptides were generated during the simulated IB passage. No peptides were identified in the basolateral compartment for fish gelatin.

**Figure 7:**
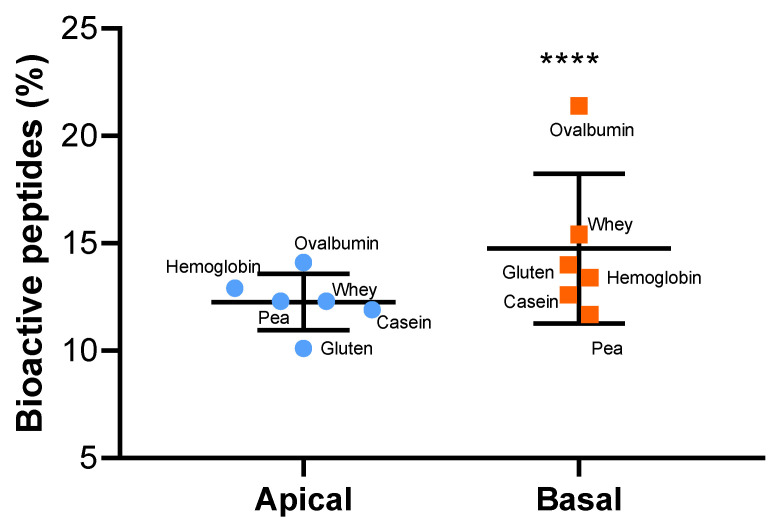
The richness in potential DPP-IV-inhibitory peptides increases during the IB transfer. The values are the percentage of bioactive peptides (with predicted DPP-IV IC_50_ < 300 µg·mL^−1^) for each experimental condition in each compartment (apical/basal). The statistical analysis was performed using a one-sample *t* test (**** *p* < 0.0001) between the basal and apical means.

**Figure 8:**
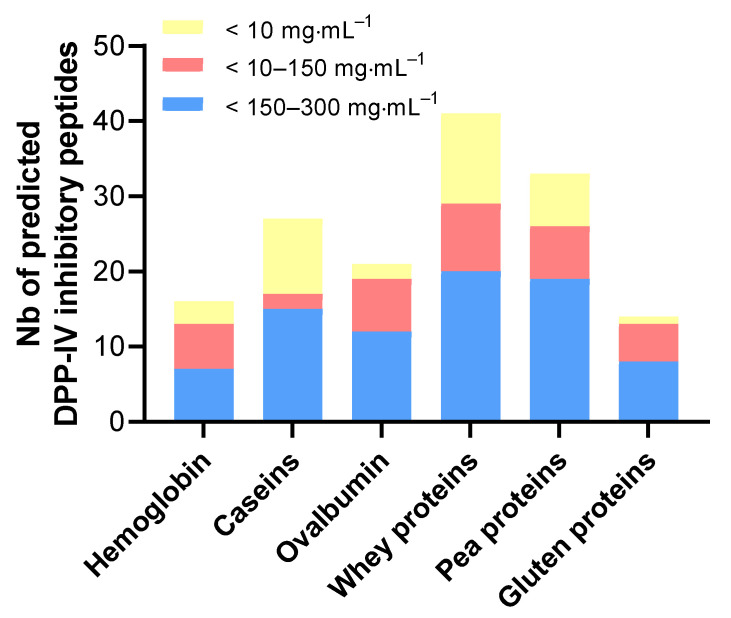
Number of QSAR-predicted DPP-IV-inhibitory peptides identified in the basal compartment after the IB transfer. The DPP-IV-inhibitory peptides (de novo included) are classified according to their DPP-IV IC_50_ values predicted with the QSAR model (IC_50_ < 10, 10–150, and 150–300 µg·mL^−1^) for each digested protein sample.

QSAR analysis was performed to predict the DPP-IV-inhibitory activity of the identified peptides [37]. Peptides from the apical and basolateral compartments with a predicted DPP-IV IC_50_ of less than 300 µg·mL^−1^ were selected (Figure 7) and classified according to their IC_50_, as assigned by the QSAR analysis (Figure 8).

Overall, from a qualitative point of view, there was a higher percentage of potential DPP-IV-inhibitory peptides (QSAR predicted DPP-IV IC_50_ < 300 µg·mL^−1^) in the basolateral (14.75%) compared to the apical compartment (12.27%) after 1.5 h of incubation.

Figure 8 represents the variety of bioactive peptides originating from all of the the digested dietary proteins with a predicted DPP-IV IC_50_ value below 300 µg·mL^−1^. Only the peptide sequences belonging to the protein fractions identified by MS were considered among all of the sequences identified upon interrogation of the databases and by the spectral reading of the de novo. Peptides from the SGID control were not included, and consequently do not appear in the peptide count. The list of predicted bioactive peptides is presented in Table S1.

Figure 8 shows that, for all protein samples, the gastrointestinal digestion and the hydrolysis occurring during the IB passage generated peptides with high predicted DPP-IV-inhibitory activity. The digestion of pea and whey proteins yielded a greater variety of DPP-IV-inhibitory peptides—approximately twice as many as that of hemoglobin, ovalbumin, and gluten proteins.

## 3. Discussion

Many studies have identified DPP-IV-inhibitory peptides in protein hydrolysates originating from milk [31,37], plants [27,38,39,40] and fish [27,41]. However, very few studies have directly compared different sources of dietary proteins for their ability to inhibit the DPP-IV activity. Moreover, differences in the protocols used for measuring DPP-IV inhibition make comparisons difficult. Currently, diverse study models are used to identify food-derived peptides that may inhibit DPP-IV activity. Most studies measure DPP-IV-inhibitory activity using purified and recombinant DDP-IV in vitro. Recently, an in vitro approach involving live intestinal epithelial Caco-2 cells was developed by our team [42], and has since been used in several studies to assess DPP-IV-inhibitory activity [33,39,42,43,44]. DPP-IV-inhibitory activity has also been measured ex vivo in rat and human plasma [13,45,46,47,48].

In this context, the objective of this study was first to compare the DPP-IV-inhibitory potential of seven different food-protein substrates both in vivo and in vitro at different levels. In the first instance, we compared the impact of the protein source on plasma DPP-IV activity in rats. It should be noted that, unfortunately, the whey and gluten protein samples were not evaluated in vivo for technical reasons (refer to Section 4.4.2). Interestingly, all of the proteins except caseins decreased plasma DPP-IV. Caseins are classified as slow-digesting proteins because of their ability to form poorly soluble structures which are prone to gelling at the low pH conditions in the stomach, thereby slowing down digestion [36]. Indeed, the plasma DPP-IV activity was measured 30 min after oral administration to fasted rats. It is likely that the caseins were not yet equally digested compared to the other proteins studied. Therefore, the intestinal uptake of casein-derived DPP-IV-inhibitory peptides was lower, potentially explaining the observed absence of DPP-IV-inhibitory activity with the caseins. In contrast, the oral administration of pea proteins led to the most potent DPP-IV inhibition. Pea proteins have previously been partially hydrolyzed by pepsin in order to facilitate oral administration. This may explain the overall higher DPP-IV-inhibitory activity observed, as partial hydrolysis can improve the digestion rate and absorption [49].

Nevertheless, the different peptides which are capable of reaching the biological target in sufficient quantity, along with the peptide sequence and length, may also explain the differences observed in plasma DPP-IV inhibition with different starting protein substrates. Indeed, the intestinal peptidomes generated during rat digestion varied in line with the substantial differences inherent to the protein sequence, conformation and digestibility of the native proteins.

These results provide new insights into the short-term effects of the oral administration of different proteins on plasma DPP-IV activity in rats. In order to further study the peripheral effects of the digested protein-derived peptides at the intestinal level, in vitro experiments using a two-cell-based model was used. DPP-IV activity was measured following simulated peptide IB transfer, and the mechanisms involved were further explored. First, the different proteins were digested in vitro, and the DPP-IV inhibition was assayed at the enterocyte level using live Caco-2 cells. The results highlighted a major impact of the protein source in the inhibition of intestinal DPP-IV activity. Indeed, after one hour of incubation, the digested hemoglobin, caseins, whey, pea, and gluten proteins showed significantly lower DPP-IV IC_50_ values than those obtained for gelatin and ovalbumin digests. The DPP-IV IC_50_ obtained in this study ranged from 1.5 to 10 mg·mL^−1^. In comparison, Caron et al. [42] found an IC_50_ for hemoglobin protein (1.6 mg·mL^−1^), which is relatively close to the IC_50_ found in this study (2.8 ± 0.4 mg·mL^−1^). Similarly, Theysgeur et al. [33] reported an IC_50_ of 3.7 mg·mL^−1^ for a hydrolyzed fish by-product, while Santos-Hernández et al. [50] found an IC_50_ value of 1.17 mg·mL^−1^ for whey protein digests, 0.87 mg·mL^−1^ for casein digests, and 1.75 mg·mL^−1^ for egg white digests using the same cell-based method. Rivero-Pino et al. [27] found an IC_50_ of 2.93 mg·mL^−1^ for pea protein using an in vitro model. Except for fish gelatin and ovalbumin, the IC_50_ values found in the literature are reasonably similar to those measured in the present study. For ovalbumin, the results seem not to be fully comparable because, in this study, we used purified albumin, while Santos-Hernández et al. used egg white protein powder, which also contains ovotransferrin, ovomucoid, ovomucin, and lysozyme [49].

An IB in vitro model using a Caco-2/HT29-MTX coculture was set up to simulate the last stages of protein digestion and absorption. Under these experimental conditions, digested hemoglobin, caseins and ovalbumin significantly reduced DPP-IV activity (by between 5 to 10%) after simulated IB passage. This does not follow the same trend as the results obtained in vivo in rats. Several assumptions could be made: (a) the human SGID and intestinal cell models may have been phylogenetically too far apart from the in vivo rat model; (b) the difference in the digestion duration between the in vivo and in vitro protocols may have influenced the results (30 min after ingestion for the in vivo results versus complete digestion (4 h) and a 1.5 h IB incubation time for the in vitro results); (c) the levels of some peptides and amino acids crossing the IB were below their limit of detection; (d) the permeability of the in vitro IB model was lower than that in physiological conditions, which significantly reduces the passage of molecules through the IB [51]; or (e) the glycaemia-reducing mechanisms linked to DPP-IV activity are much more complex than what could be achieved with the in vitro model.

Nevertheless, the original model set up herein gave us the ability to study the different mechanisms occurring during IB passage, such as DPP-IV gene expression regulation. Indeed, digested plant proteins (pea and gluten), hemoglobin, and gelatin decreased DPP-IV gene expression. These results are in accordance with those of Domenger et al., who had previously shown a tendency for digested hemoglobin-derived synthetic peptides to decrease DPP-IV mRNA expression after 24 h incubation with Caco-2 cells [52]. However, to our knowledge, this is the first time that the regulation of DPP-IV gene expression has been reported with digested proteins.

The effects of digested protein on IB permeability and integrity were assessed by measuring the TEER and the apparent permeability (P_app_). Moreover, the expression of ZO-1, occludin, and claudin 1, 2, 4, 12 and 15 genes—which participate in tissue integrity, cell maintenance, shape, and the passage of electrolytes and small molecules—was assayed [53]. A decrease in the TEER was observed after 1.5 h of contact with digested whey, hemoglobin and caseins, which could be linked to the decrease in ZO-1, occludin, and claudin 1 and 4 gene expression. Other mechanisms may be involved for digested whey proteins, as no modulation in TJ gene expression was observed. Moreover, digested gluten decreased the expression of ZO-1, occludin and claudin 1 without affecting the TEER. However, it is essential to specify that even though digested caseins and hemoglobin decrease TEER, this remains within acceptable P_app_ values and below the control condition value (4.15 × 10^−6^ ± 7.88 × 10^−7^ cm·s^−1^). The P_app_ is used to evaluate passive drug absorption by directly assessing the permeability through Caco-2/HT29-MTX. Even if there is no reference value for the Caco-2/HT-29 MTX coculture model, the P_app_ obtained was within the acceptable limit values. The P_app_ is considered correct for the Caco-2 cell monolayer if it is less than 1 × 10^−6^, and HT-29 cells are known to be much more permeable compared to Caco-2 cells [54,55].

Thus, all of the digested proteins preserve TJ integrity. Nevertheless, all of the digested proteins did not similarly affect IB permeability. The P_app_ of ovalbumin (1.03 × 10^−6^ ± 2.77 × 10^−7^ cm·s^−1^) was lower than that of the control (4.15 × 10^−6^ ± 7.88 × 10^−7^ cm·s^−1^). Likewise, the P_app_ of fish gelatin (8.70 × 10^−7^ ± 1.79 × 10^−7^) was lower than that of the control, suggesting that ovalbumin and fish gelatin improve the integrity of the TJ complex. However, P_app_ is only an indicator of passive diffusion, and the passage of peptides through the IB can follow different pathways. The uptake of amino acids and di- and tripeptides also involves passive diffusion, endocytosis, transcellular carrier-mediated transport, or some transmembrane co-transporters such as the proton-dependent H+/peptide PepT1 and the sodium-dependent oligopeptide transporters SOPT1 and SOPT2 [56].

Regarding the effect of digested proteins on TJ gene expression, the decrease in ZO-1, occludin, and claudin 1 and 4 at transcript levels observed with digested hemoglobin, casein, and gluten proteins appears surprising. A study by Yasumatsu and Tanabe in 2010 showed that peptides derived from α_S2_-casein up-regulated occludin gene expression, increased the TEER, and then enforced the tight junction barrier [57]. Another study by Anderson et al., in 2019, showed that a bovine colostrum protein concentrate (not digested) increased the epithelial barrier integrity of the Caco-2 cell monolayer [58]. Bavaro et al., in 2021, showed that the heat treatment of infant milk affected the integrity of the IB vs. microfiltered infant milk, with TJ proteins and the claudin 1 gene expression being significantly higher with microfiltered infant milk [59].

In addition, peptide identification by the proteomics approach with QSAR analysis was employed for the bioactivity prediction of sequences identified at the apical and basolateral side of the IB model. The number of peptides found in both the apical and basal compartments differed depending on the starting protein substrate. Among these peptide populations, some peptides identified at the apical side were also found at the basolateral side, while new peptides were only identified at the basolateral side. This suggests that some of the peptides were resistant to IB peptidases, and that some new peptides were generated during the IB passage. Three times more peptide sequences were identified in the apical compartment vs. the basal compartment, further highlighting the IB barrier integrity and peptide selectivity, as previously described [59]. Indeed, it is well known that di- and tri-peptides cross the IB more easily than larger peptide sequences or free amino acids. Peptide permeation is mediated by specific transporters such as PepT1 [60]. In addition, selected short peptide sequences can exert good DPP-IV-inhibitory activity. Unfortunately, these short peptides could not be identified in the present study, as it is still very challenging to identify short peptide sequences (<5 AA residues) by MS in digested samples [61,62].

Regarding the fish gelatin source, no peptides could be identified in the IB basal compartment, and very few peptides were identified with the proteomics analysis. The difficulties of analysing fish gelatin are linked to (i) the gelling nature of gelatin making protein extraction difficult, (ii) the high number of specific post-translational modifications (proline hydroxylation), and (iii) the lack of fish protein database annotation.

The QSAR analysis permitted the calculation of a predicted DPP-IV IC_50_ value for each identified peptide. Based on the results, a higher proportion of bioactive peptides appeared to be present in the basal compartment. The passage of peptides through the IB seemed to generate new inhibitory peptides, even though short peptides were not identified in our study. This information is essential because it further highlights the impact of IB peptidases on DPP-IV-inhibitory peptide generation during peptide transfer. Some peptides are hydrolyzed by intracellular peptidases or by peptidases located at the basal pole of the IB. The variety of peptides generated during digestion is dictated by the starting protein sequences. The different DPP-IV-inhibitory effects observed in vitro and in vivo in this study originate from the overall pool of peptides which are released during digestion and capable of crossing the IB.

The in silico results have been used as predictions, and they should therefore be taken with caution. In silico tools do not always have a high predictive capacity because they do not always translate in an adequate correspondence between the predicted and IC_50_ value. Indeed, QSAR analyses only use a small fraction of the primary peptide sequence, which relies on peptide motifs that have previously been reported as being important for specific bioactive properties. Moreover, the predictive approach used herein remains qualitative due to the impossibility of analysing the complexity of the digested samples and evaluating each peptide in relation to its sequence and abundance. Nevertheless, this study provides essential information regarding the diversity and nature of the potential DPP-IV-inhibitory peptides depending on the parent dietary protein substrate.

## 4. Materials and Methods

### 4.1. Protein Sample Substrates

The bovine hemoglobin (H2625), chicken egg white ovalbumin (A5503), and wheat gluten (G5004) powders came from Sigma-Aldrich (St. Louis, MO, USA). The whey protein isolate (Promilk^®^ 852FB) and native micellar caseins (Prodiet^®^ 85B) powders, extracted by a specific membrane separation and spray-drying process, were from Ingredia (Arras, France). The pea protein powder (Nutralys^®^ S85F) came from Roquette, and the fish gelatin (high-molecular-weight dried cold fish gelatin) was from Kenney & Ross. The total nitrogen content of the powders was evaluated by the Kjeldahl method [63,64], and was converted to the protein content with the appropriate conversion factors.

### 4.2. Chemicals

The following chemicals were obtained from Sigma-Aldrich (St. Louis, MO, USA): porcine pepsin (P6887), pancreatin from porcine pancreas (P1750), Gly-Pro-7-amido-4-methylcoumarin hydrobromide (H-Gly-Pro-AMC, HBr), Dulbecco’s modified Eagle’s medium (DMEM), L-glutamine, penicillin and streptomycin. The foetal bovine serum (FBS) was from GIBCO Invitrogen (Karlsruhe, Germany). The water was purified using the Milli-Q system (Millipore, Burlington, NJ, USA).

### 4.3. One-Dimensional Sodium Dodecyl Sulfate-Polyacrylamide Gel Electrophoresis (SDS-PAGE)

Each protein substrate was analyzed by SDS-PAGE following the Laemmli method [65]. In brief, the protein substrates were solubilized in H_2_O at 1 (for hemoglobin and ovalbumin), 4 (for caseins, whey proteins, fish gelatin and pea proteins) and 30 mg·mL^−1^ (for gluten), and were diluted in Laemmli buffer containing β-mercaptoethanol and SDS before heating at 95 °C for 10 min. Then, 24 µL samples and 4 µL molecular mass marker solution (Precision Plus Protein^TM^ Standards, Bio-Rad, Marnes-la-Coquette, France) were deposited on an Any-kD^TM^ Mini-Protean^®^ TGX Stain-free^TM^ gel (Bio-Rad). Protein migration was performed for 1 h in a buffer containing Tris-base (25 mM), glycine (0.19 mM), and SDS (3.5 mM) at constant voltage of 120 V. Thereafter, fluorescence generated by reaction between the gel-trihalo compounds and tryptophan residues of the proteins was revealed after 5 min of activation time with Gel Doc^TM^ XR+ and Image Lab 6.1.0 software (Bio-Rad).

### 4.4. In Vivo Experiment

#### 4.4.1. Animal Conditions

The animal experiments were carried out according to the French ethical guidelines for studies on experimental animals (Animal house agreement no. 5900912, Authorization for Animal Experimentation no. 20992-201906031147321 v3, project approved by the local ethical committee no. CEEA75). All precautions were taken to prevent any potential animal suffering. In total, 64 12–week-old Wistar male rats were purchased from Envigo (Gannat, France), and were housed in individual cages during the experiment. The temperature, humidity, and light–dark cycle were controlled, and the rats were fed ad libitum with a standard rodent diet (Serlab, 3430PMS10). The rats had a week of acclimatization, followed by three days of force-feeding training with water. Before starting the experiment, the rats were weighed and placed randomly in 8 groups (n = 8).

#### 4.4.2. Animal Experiment

The rats were fasted for 16 h before the experiment. On the experiment day, the rats were force-fed using an intragastric tube with a 100 mg·mL^−1^ protein solution, except for the gluten and whey protein samples because their solubility was too low for force-feeding. The pea protein sample was partially hydrolyzed with pepsin in order for it to be administered. The volume of solution given was adjusted to the weight of each rat at 1 g·kg^−1^. The rats were sacrificed 30 min after the gavage, and blood samples were collected in tubes containing 10 µL 5% ethylenediamine tetraacetic acid (EDTA). The tubes were centrifuged, and the supernatants were stored at −80 °C before being assayed for plasma DPP-IV activity.

### 4.5. In Vitro Protein SGID

Before starting the in vitro experiments, protein powders were digested according to the INFOGEST harmonized protocol [35] adapted for protein alone [36]. In brief, 2 g protein was solubilized in 8 mL ultrapure water and mixed with 8 mL simulated salivary fluid at pH 7.0 for 2 min. Then, a 4 mL salivary sample was collected and 12 mL simulated gastric fluid containing pepsin (6500 U·mL^−1^) was added to the batch and incubated for two hours at pH 3.0. In the same way, a 4 mL gastric sample was collected at the end of the incubation time and heated at 95 °C for 10 min to inactivate the pepsin. In total, 20 mL simulated intestinal fluid containing pancreatin (45 U·mL^−1^) was finally added to the batch and incubated for two hours at pH 7.0. At the end of the incubation time, the intestinal samples were heated at 95 °C for 10 min and centrifuged at 13,400× *g* for 10 min at room temperature; then, supernatants were collected and frozen at −20 °C for further analysis. A control blank SGID was performed in the same conditions but without protein. The protein digestion was repeated three times.

### 4.6. Cell Lines and the Culture Routine

Caco-2 cells and mucus-secreting HT29-MTX cells, both from human colon carcinoma, were purchased from Sigma-Aldrich (Villefranche-sur-Saône, France). These two cell lines were routinely cultivated in 75 cm^2^ flasks (Sarstedt, Nümbrecht, Germany), under 5% CO_2_, at 37 °C, in DMEM (PAN Biotech, Aidencach, Germany) supplemented with penicillin/streptomycin (100 U·mL^−1^), 2 mM L-glutamine, and 10% heat-inactivated FBS.

### 4.7. Intestinal Barrier (IB) Passage

#### 4.7.1. IB Model

A Caco-2/HT29-MTX coculture model was used to analyze the permeation of digested proteins through the IB. Caco-2 cells and HT29-MTX cells were seeded on inserts at a ratio of 90/10 and at a density of 20,000 cells per transwell (microporous PET membrane, 3 μm pore size, Corning, Glendale, CA, USA). Then, the inserts were incubated for 21 days to allow the growth and differentiation of the coculture, under 5% CO_2_, at 37 °C, in DMEM (PAN Biotech, Aidencach, Germany) supplemented with penicillin/streptomycin (100 U·mL^−1^), 2 mM L-glutamine, and 10% heat-inactivated FBS. The culture medium was changed twice a week until the experimentation day. The Caco-2/HT29-MTX coculture monolayer was checked at day 21 (Figure S1).

A simpler IB model was also carried out with Caco-2 cells only. This model was used to measure the DPP-IV activity of intestinal epithelial cells. Caco-2 cells were seeded in a 96-well black plate at a density of 8000 cells per well, in a volume of 150 μL supplemented DMEM. The plates were incubated for 15 days before experimentation.

#### 4.7.2. IB Permeation Experiment

On the day of the permeation experiment, inserts of Caco2/HT29-MTX cells were washed with phosphate buffer saline (PBS), and 500 μL 31.25 mg·mL^−1^ digested proteins were deposited in the apical compartment, whereas 1 mL non-supplemented DMEM was placed in the basal compartment. After 1.5 h incubation, the apical and basal media were collected and frozen at −20 °C until further analysis.

#### 4.7.3. IB Integrity Analysis

The IB integrity was evaluated before and after the incubation of the digested proteins with the cells by measuring the TEER. The paracellular permeability of the Caco-2/HT29-MTX cell monolayer was determined using Lucifer yellow (LY) directly after the permeation experiment. The transwell inserts were thus emptied, and Hank’s balanced salt solution (HBSS) containing 100 µM LY was added into the apical chamber. The same solution without LY was added into the basal chamber. Then, the transwells were incubated for 90 min, and basal samples were collected at 15, 30, 45, 60 and 90 min; the fluorescence was measured with a spectrofluorometer (Spectramax, Molecular Devices, San José, CA, USA) at 530 nm (excitation) and 585 nm (emission). The apparent permeability coefficient (P_app_) was then calculated according to the following equation:

$$\mathrm{Papp}\;\left(\frac{\mathrm{cm}}{s}\right)=\frac{1}{S*C0}*\frac{\mathrm{dQt}}{\mathrm{dt}}$$

where S is the surface area of the membrane, C_0_ is the initial concentration of LY in the apical compartment, and Q is the amount of LY transported from the apical to the basal chamber in a specific time period (t = 15, 30, 45, 60 or 90 min).

### 4.8. DPP-IV Activity Assay

#### 4.8.1. Plasma DPP-IV Activity Assay

The activity of DPP-IV in rat plasma was assessed by adding 10 µL plasma to 140 µL 1 mM Gly-Pro-AMC diluted in Tris-HCl (pH 8.0) buffer. Then, the fluorescence (360 nm excitation, 438 nm emission) was recorded every 2 min for 1 h at 37 °C using 96-well black microplates and a Xenius-XC spectrofluorometer (Safas Monaco, Monaco). The slope was then calculated and used to determine the percentage of DPP-IV activity inhibition of the different digested proteins compared to the control (water group).

#### 4.8.2. In Vitro Intestinal DPP-IV Activity Inhibition Assay

In order to measure the DPP-IV activity before IB passage at the enterocyte level, analysis was performed following the method described by Caron et al., 2017, with some adjustments [42]. In brief, Caco-2 cells were washed with PBS, and digested proteins diluted in pH 7.4 PBS at four concentrations were deposited on Caco-2 cells with Gly-Pro-AMC fluorescent substrate at 1 mM. The kinetics were monitored with the same parameters as in vivo DPP-IV activity analysis. Then, IC_50_ for each digested protein was calculated using the slope. In order to measure the in vitro intestinal DPP-IV activity after in vitro IB permeation, 50 μL Gly-Pro-AMC substrate (1 mM) and 25 μL DPP-IV human recombinant enzyme (0.018 U·mL^−1^) were added to 100 μL basolateral medium. The slopes were then calculated and used to determine the percentage of DPP-IV-inhibitory activity of the different proteins compared to the control (buffer).

### 4.9. Tight Junction Proteins and DPP-IV Gene Expression

For the gene expression experiments, Caco-2/HT29-MTX cells were seeded into 24-well plates at a density of 40,000 cells per well, with 500 μL supplemented DMEM. After 15 days of differentiation, the cells were washed with PBS, and 500 μL digested proteins (5 mg·mL^−1^) diluted in non-supplemented DMEM were added; the cells were incubated for 2, 4 or 24 h under 5% CO_2_ at 37 °C. At the end of the contact, the supernatant was removed, and RNA was extracted using the modified NucleoZOL (Macherey-Nagel, Düren, Germany) protocol. First, the RNA concentration and purity were assessed using the Nanodrop lite spectrophotometer (ThermoFisher scientific, Waltham, MA, USA). The reverse transcription was then performed using a RevertAid H Minus First Strand cDNA Synthesis Kit (Thermo Scientific), and the mRNA levels were determined by qPCR on a CFX Connect Real-Time PCR detection system (Biorad) using the Takyon™ No Rox SYBR^®^ MasterMix dTTP Blue (Eurogentec) and specific primers (Table 3).

### 4.10. Proteomics and Peptidomics Analysis by RP-HPLC-MS/MS and Bioinformatics

#### 4.10.1. Sample Preparations

For the characterization of the protein powders, 200 µg (for caseins, whey proteins, ovalbumin, fish gelatin, pea proteins and gluten) and 100 µg (for hemoglobin) were subjected to reduction (5 mM dithiothreitol, 5 min at 80 °C) and alkylation (3 mM iodoacetamide, 20 min at 20 °C) in 30 µL 25 mM ammonium bicarbonate buffer before overnight trypsin hydrolysis with 0.2 µg MS Grade Trypsin/Lys-C mix (Promega, Lyon, France). The digested proteins were directly analyzed by RP(C18)-HPLC-MS/MS after centrifugation (10 min at 8000× *g*).

For the peptide identification before or after IB passage, 1.5 mL apical and basal medium after 120 min of transport studies were dried using a centrifugal evaporator (miVac, Gene Vac, Ipswich, Royaume-Uni) for 2 h at 40 °C. The samples were solubilized in 50 µL 0.1% formic acid/99.9% water (*v*/*v*) (solvent A) and centrifuged (10 min at 8000× *g*). The supernatants were directly analyzed by RP(C18AQ)-HPLC-MS/MS.

#### 4.10.2. RP-HPLC-MS/MS Analyses

In total, 10 µL of the samples was chromatographically separated at 30 °C in RP-HPLC using a C18 or a C18-AQ column (150 × 2.6 mm, 3 µm particles, Interchim, Montluçon, France) on a biocompatible ACQUITY UHPLC system (Waters, Manchester, UK). For the C18 column, the acetonitrile gradient (flow rate 0.5 mL·min^−1^) was as follows: 1% solvent B (0.1% formic acid/99.9% acetonitrile (*v*/*v*)) for 1.5 min, then 1% to 45% solvent B for 43.5 min, then 45% to 70% solvent B for 5 min, followed by washing and equilibrating procedures with, respectively, 95% and 1% solvent B for 5 min each. For the C18-AQ column, the acetonitrile gradient (flow rate 0.5 mL·min^−1^) was as follows: 1% solvent B for 5 min, then 1% to 10% solvent B for 25 min, then 10% to 30% solvent B for 20 min, followed by washing and equilibrating procedures with, respectively, 95% and 1% solvent B for 5 min each. The HPLC eluent was then directly electrosprayed at the end of the column at a voltage of 3 kV, using a desolvation gas (N_2_) at a flow of 600 L·h^−1^, a nebulizer gas flow of 2.5 bar, and a temperature of 300 °C. The HPLC was coupled with a SYNAPT-G2-Si mass spectrometer (Waters) that was previously calibrated using a sodium formate solution. The MS measurements were made in sensitivity- and positive-mode using proprietary MassLynx software (version 4.1, Waters). The MS and MS/MS analyses were performed in data-dependent analysis (DDA) mode, and mass data were collected in the measurement range of 50 to 1700 m/z using lock mass correction with 556.632 m/z, corresponding to simply charged leucine enkephalin. A maximum of 10 precursor ions with an intensity threshold of 10,000 counts were selected to be fragmented by collision-induced dissociation (CID) at an energy collision of 8 V to 9 V for low-molecular-mass ions, and at a range of 40 V to 90 V for high-molecular-mass ions. The RP-HPLC-MS/MS analysis was carried out in triplicate for each protein substrate on three independent SGID (for basal compartment) and the transport study (for apical and basal compartment).

#### 4.10.3. Peptide Identification

Database searches were performed on PEAKS^®^ Studio X+ (Bioinformatics Solutions Inc., Waterloo, ON, Canada) using the UniProt database (access online, July 2020) for blank digestion, and the UniProt database restricted to the complete proteome of the *Bos taurus* organism (access on line, April 2019) for cow and dairy sources, that of the *Gallus gallus* organism (access on line, February 2021) for egg sources, that of the *Pisum sativum* organism (access on line, September 2020) for pea sources, that of Triticum (acess online, October 2020) for wheat sources, and restricted to collagen proteins (access online, March 2020) for fish sources. A mass tolerance of 35 pp, 3 missed cleavage sites for trypsin hydrolysis (for protein source characterization) or no specific enzyme (for the transport study), and an MS/MS tolerance of 0.2 Da were allowed. Variable methionine oxidation was also considered. Peptide sequences identified by PEAKS^®^ Studio X+ were filtered with a false discovery rate (FDR) < 1%, while peptide sequences identified by de novo processing (for transport study) were filtered according to an ALC > 80%.

#### 4.10.4. QSAR of DPP-IV-Inhibitory Peptides

The peptides identified by database confrontation or de novo identification were sorted according to their actual occurrence within the sequence of the identified protein fractions. Then, peptides with the following features were selected: Phenylalanine, Isoleucine, Leucine or Tryptophane at the N-Terminal position, Proline or Alanine in the P2 position, or Proline at the C-Terminal position. The determination of a predicted DPP-IV IC_50_ value was carried out with the QSAR model developed by Nongonierma and FitzGerald (2016) [66] and the structural v-scale from Lin et al. [67]. The peptide sequences incorporated in the QSAR model had a length ranging from 2 to 35 amino acid residues. 

### 4.11. Statistical Analysis

The statistical analysis was carried out using GraphPad Prism 8 software. A Shapiro–Wilk test was systematically performed in order to verify the normal distribution of the values. One-way or two-way ANOVA was then used, followed by a Tukey or a Dunnett test for mean comparisons. When the values did not follow a normal distribution, a Kruskal–Wallis test was performed, followed by a Dunn test for pairwise comparison. The differences were considered significant for a *p*-value < 0.05. The values were expressed as mean ± SEM.

## 5. Conclusions

For the first time, the effects of dietary proteins originating from different sources on the DPP-IV-inhibitory activity were evaluated in vivo and in vitro at different intestinal levels (i.e., before and after IB permeation). This study demonstrated that the ingestion of proteins influenced DPP-IV activity in physiological conditions. Furthermore, the protein origin led to a more or less pronounced effect on the overall inhibitory properties. In parallel, this study is the first report highlighting the DPP-IV-inhibitory activity and barrier integrity protein gene expression of digested proteins in an in vitro Caco-2/HT29-MTX cell model. Furthermore, MS-based peptidome identification associated with QSAR analysis showed that peptides crossing or generated during IB transfer could inhibit DPP-IV activity differently. The DDP-IV inhibitory activity depended on the source of the starting protein substrate. Based on these results and a score attribution (Table 4), plant proteins and hemoglobin seem to be the most promising starting proteins. This study provides some insights on the elaboration of specialised diets in the framework of a personalized nutrition to prevent or manage T2D.

## Figures and Tables

**Table 1:** Protein composition of the raw protein samples determined by proteomics analysis.

Protein Sources	Identified Proteins	Number of Identified Peptides by Protein	Average Protein Molecular Mass (kDa)	Identification Score (−10 lgP)
Hemoglobin	Hemoglobin sub unit apha	47	15.2	412
	Hemoglobin sub unit beta	46	15.9	394
	Carbonic anydrase 2	3	29.1	123
	Peroxxiredoxin-2	3	21.9	112
	Serum albumin	2	69.2	72
Caseins	α_S1_-Casein	64	24.5	425
	β-Casein	53	25.1	405
	α_S2_-Casein	44	26	377
	κ-Casein	41	21.3	361
	β-Lactoglobulin	33	19.8	382
	Serum albumin	32	69.2	310
	α-Lactalbumin	10	16.2	247
	Lactadherin	11	47.4	212
	Other proteins	54	14.7 to 146.7	90 to 249
Ovalbumin	Ovalbumin	203	42.8	353
Whey proteins	β-Lactoglobulin	106	19.9	517
	α-Lactalbumin	38	16.2	387
	Serum albumin	50	69.2	366
	α_S1_-Casein	24	24.5	313
	β-Casein	19	25.1	279
	α_S2_-Casein	17	26	225
	κ-Casein	8	21.3	231
	Other proteins	116	127	116 to 300
Fish gelatin	Fibrillar collagen	11	85.6 to 127	76 to 98
	Collagenα	5	135.6	85
	Other proteins	6	125.9	79
Pea proteins	Legumin	184	7 to 64.8	185 to 447
	Vicilin	253	49.4 to 52.2	409 to 447
	Convicilin	283	62.0 to 72.1	390 to 430
	Provicilin	109	31.5 to 46.3	336 to 406
	Albumin-1/-2	33	13.9 to 26.2	105 to 347
	Lectin	18	30.2	222
	Other proteins	156	11.4 to 97.2	95 to 314
Gluten proteins	Gliadin	166	32.5 to 33.9	170
	β-amylases	120	58.9 to 62.6	238 to 272
	Glutenin	103	69.5 to 84.0	179 to 222
	Triticin	20	64.9	221
	Other proteins	40	15.5 to 65	167 to 221

Proteins were identified by proteomics analysis after the trypsin proteolysis of the protein samples, RP-HPLC-MS/MS analysis, and the bioinformatics management of MS-data. The proteins were sorted according to their PEAKS^®^ Studio identification score (X+ score; −10 lgP).

**Table 2:** Digested proteins modulate the permeability and IB integrity differently.

Intestinal Samples	P_app_ (cm·s^−1^)	ΔTEER (Ω)
		(TEER Final-TEER Initial)
Buffer control	4.15 × 10^−6^ ± 7.88 × 10^−7^	14.27 ± 68.9
Blk SGID	5.48 × 10^−7^ ± 1.48 × 10^−7^ ****	10.63 ± 8.6
Hemoglobin	2.27 × 10^−6^ ± 3.59 × 10^−7^	−241.08 ± 18.5 **
Caseins	1.75 × 10^−6^ ± 3.78 × 10^−7^	−182.00 ± 80.9 **
Ovalbumin	1.03 × 10^−6^ ± 2.77 × 10^−7^ **	83.69 ± 36.5
Whey proteins	2.12 × 10^−6^ ± 5.05 × 10^−7^	−318.31 ± 90.6 ****
Fish gelatin	8.70 × 10^−7^ ± 1,79 × 10^−7^ **	−14.38 ± 5.3
Pea proteins	1.75 × 10^−6^ ± 3.25 × 10^−7^	−119.15 ± 61.1
Gluten proteins	1.78 × 10^−6^ ± 3.89 × 10^−7^	23.15 ± 74.0

**Table 3:** Primers used for the qPCR experiments.

	Genes	Forward Primer	Reverse Primers
*Homo sapiens* origin	Hypoxanthine phosphoribosyltransferase 1	GCCCTGGCGTCGTGATTAGT	GCAAGACGTTCAGTCCTGTCC
	Dipeptidyl peptidase 4	ACAGAATCACATGGACGGGG	CGTTTGGAGACCACCACAGA
	ZO-1	CGGTCCTCTGAGCCTGTAAG	GGATCTACATGCGACGACAA
	Occludin	CAGGGAATATCCACCTATCACTTCAG	ATCAGCAGCAGCCATGTACTCTTCAC
	Claudin 1	CCCTATGACCCCAGTCAATGC	GGATAGGGCCTTGGTGTTGG
	Claudin 2	TGGCCTCTCTTGGCCTCCAACTTGT	TTGACCAGGCCTTGGAGAGCTC
	Claudin 4	CCACTCGGACAACTTCCCAA	ACTTCCGTCCCTCCCCAATA
	Claudin 12	CTGAGAGGGAGACGCTCCAA	GTACCTGACAGTTCCAAAACAGC
	Claudin 15	GTACCCCGGAACCAAGTACG	CGTTTCTGCCGTATTTGCCA

**Table 4:** Global assessment of the DDP-IV-inhibitory potential of protein from different origins.

	Hemoglobin	Caseins	Ovalbumin	Whey Proteins	Fish Gelatin	Pea Proteins	Gluten Proteins	SGID blk
In vivo DPP-IV inhibition	1	0	3		3	4		0
In vitro IB Apical DPP-IV inhibition	4	4	0	4	0	4	4	-
In vitro IB basal DPP-IV inhibition	4	3	2	1	1	0	0	0
In vitro DPP4 gene expression inhibition	2	0	0	0	2	2	4	2
**SCORE**	**11**	**7**	**5**	5 *	**6**	**10**	8 *	**2**

Scores were assigned based on significant differences (*p* < 0.0001 = 4 points; *p* < 0.001 = 3 points; *p* < 0.01 = 2 points, and *p* < 0.05 = 1 point) in different experiments. Grey cases and * means that proteins have not been studied in vivo.

## Data Availability

The data were generated during the study.

## Supplementary Materials

The following supporting information can be downloaded at: https://www.mdpi.com/article/10.3390/ijms23158365/s1.
