# Supplementary material for: In Vivo and In Vitro Comparison of the DPP-IV Inhibitory Potential of Food Proteins from Different Origins after Gastrointestinal Digestion
Source: Int J Mol Sci. 2022 Jul 28;23(15):8365. doi: 10.3390/ijms23158365 (PMC9369239; doi:10.3390/ijms23158365)
Supplement: Supplementary file 1 [file ijms-23-08365-s001.zip › ijms-1813128-supplementary.pdf]

International Journal of
*Molecular Sciences*

MDPI

**Table S1.** Peptide sequences identified by the peptidomics approach (RP-HPLC-MS/MS and the bioinformatic retreatment of MS data), and the predicted DPP-IV $IC_{50}$ value obtained by the QSAR analysis.

| Substrate | Peptide sequence | Predicted DPP-IV $IC_{50}$ ($\mu g.mL^{-1}$) |
|---|---|---|
| **Hemoglobin** | LVVYPWT | too small |
| | FAED | too small |
| | YPWT | too small |
| | LLGN | too small |
| | VVYPWT | too small |
| | VLDS | too small |
| | LLGNV | too small |
| | FTPA | 23.60 |
| | CFS | 25.41 |
| | WH | 46.32 |
| | LLVVYP | 48.05 |
| | HLDD | 125.24 |
| | HVDPEN | 147.65 |
| | LVVYP | 159.59 |
| | LIVTQTMKGLDIQKVAGTWYSLA-MAASDISLLDAQSAPLR | 175.04 |
| | FGDL | 267.09 |
| **Caseins** | WC | too small |
| | WL | too small |
| | FPK | too small |
| | LVYPFPGPIPN | too small |
| | LVYPFPGPIHN | too small |
| | FPEV | too small |
| | LLS | too small |
| | FLPYP | too small |
| | YPFPGPIPN | too small |
| | FPM | too small |
| | YPE | too small |
| | LFC | too small |
| | YAEA | too small |
| | LPH | too small |
| | LPQ | 5.59 |
| | YFYPE | 38.70 |
| | VPN | 146.00 |
| | FM | 153.44 |
| | LVYPFPGPI | 167.05 |
| | LVYPFPGPI | 167.05 |
| | LIVTQTMKGLDIQKVAGTWYSLA-MAASDISLLDAQSAPLR | 175.04 |
| | TLTDVEN | 192.62 |
| | LPYP | 223.65 |

| | | |
|---|---|---|
| | WQ | 233.69 |
| | YM | 246.95 |
| | LPPL | 288.37 |
| | LIDIGVAG | 288.84 |
| **Ovalbumin** | ILELPFASGTMS | too small |
| | ILELPFASGT | too small |
| | LLPDE | too small |
| | VLLPDEVSGLEQLES | too small |
| | LLPDEV | too small |
| | LPE | too small |
| | VLLPD | too small |
| | VLQPS | too small |
| | LPDE | too small |
| | LVLLPDEV | too small |
| | VLQPSSVDS | too small |
| | LPDEV | too small |
| | VLLPDEVSGLEQLESIINFEK | 44.63 |
| | ILELPFASGTM | 45.40 |
| | VLLPDEVSGLEQLESIINFE | 101.70 |
| | ISQAVHA | 113.91 |
| | LPR | 125.42 |
| | ILELPFASGTMSM | 133.16 |
| | VLLPDE | 139.70 |
| | LLPDEVSG | 200.06 |
| | VLLPDEV | 227.83 |
| **Whey protein** | WL | too small |
| | FLDDDLTDD | too small |
| | WT | too small |
| | FPE | too small |
| | LIVTQTMKGLDIQKVAGTWYSLA-MAASDISLLDAQSAPLRVYVEELKPTPEGDLEILLQK | too small |
| | LGYS | too small |
| | LVYPFPGPIPN | too small |
| | LLD | too small |
| | LLS | too small |
| | LVNE | too small |
| | LPE | too small |
| | YVEELKPTPE | too small |
| | FLDDDLTDDI | too small |
| | LVLDTD | too small |
| | LVTD | too small |
| | FLDDDL | too small |
| | LPH | too small |
| | LPLS | too small |
| | CLLA | too small |
| | LPQ | 5.59 |
| | FLDDDLTDDIM | 16.09 |
| | LLK | 25.24 |

| | | |
|---|---|---|
| | LLH | 30.76 |
| | FME | 40.58 |
| | WH | 46.32 |
| | CLME | 62.53 |
| | YVEELKPTPEGDLE | 117.33 |
| | LPV | 119.59 |
| | LPM | 125.76 |
| | LPMH | 156.54 |
| | VLDTD | 157.00 |
| | LVYPFPGPI | 167.05 |
| | LIVTQTMKGLDIQKVAGTWYSLA-MAASDISLLDAQSAPLR | 175.04 |
| | YVE | 187.83 |
| | LLLV | 208.44 |
| | LPYP | 223.65 |
| | WQ | 233.69 |
| | VPPE | 240.28 |
| | LPPL | 288.37 |
| | DLKA | 293.93 |
| **Pea proteins** | WM | too small |
| | YLGGNPET | too small |
| | FVPH | too small |
| | LLYV | too small |
| | LFEN | too small |
| | LLPH | too small |
| | MLPH | too small |
| | LLSGN | too small |
| | LLENQ | too small |
| | VFKT | too small |
| | LLTT | too small |
| | LVEV | too small |
| | LPH | too small |
| | FLM | too small |
| | VLFA | too small |
| | FVSK | too small |
| | LFE | too small |
| | LAKV | too small |
| | LPQ | 5.59 |
| | VLASLPT | 41.17 |
| | WD | 97.96 |
| | LGPK | 108.85 |
| | LPR | 125.42 |
| | LFLPQ | 127.37 |
| | VPN | 146.00 |
| | LLFL | 149.78 |
| | FM | 153.44 |
| | FPF | 167.94 |
| | YVE | 187.83 |

| | | |
|---|---|---|
| | LGGC | 215.87 |
| | MLM | 238.78 |
| | YM | 246.95 |
| | TLDYWPS | 251.18 |
| **Gluten proteins** | WP | too small |
| | YVFD | too small |
| | FPQ | too small |
| | LPT | too small |
| | FGMA | too small |
| | LLPR | too small |
| | VLFA | too small |
| | LPQ | 5.59 |
| | LLAA | 28.51 |
| | YPTSPQ | 87.23 |
| | WD | 97.96 |
| | LPV | 119.59 |
| | LPR | 125.42 |
| | FPF | 167.94 |

Predicted DPP-IV $IC_{50}$ values were calculated according to the v-scale and the QSAR model [66]. The DPP-IV IC 50 values below 0 are marked as 'too small', and are assumed to be very efficient inhibitors.

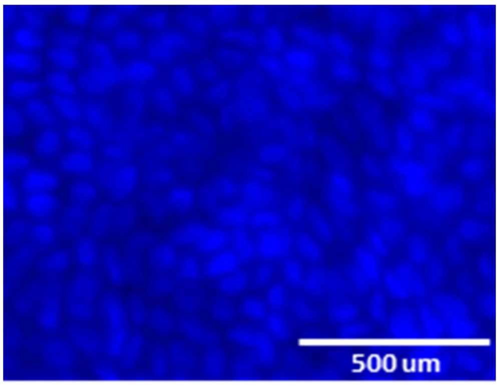


**Figure S1.** Caco-2/HT29-MTX monolayer after 21 days of culturing.

Fluorescent microphotography of a Caco2/HT29-MTX differentiated-cell monolayer after 21 days of culturing. The cells were seeded at day 0 on inserts at a Caco-2/HT29-MTX ratio of 90/10, and at a density of 20,000 cells per $cm^2$. The insert was washed with PBS and cut up to be incubated for fifteen minutes in the dark with NucBlue Live Cell Stain ReadyProbes reagent (Invitrogen, Waltham (MA), USA). Photographs were taken with an Evos FL microscope (Life Technologies, Carlsbad (CA), USA). The confluence of the cells was reached after 7 days of culturing, and the differentiation continued for about 14 days. The TEER mean values obtained during the differentiation were about 80, 310, 520, and between 750 and 1000 Ω.cm-2 after 9, 12, 16 and 21 days of culturing, respectively.
